# Total Gastrectomy for Gastric Malignancy: Trends Over 15 Years in Major Morbidity, Mortality, and Patient Selection From The National Surgical Quality Improvement Program

**DOI:** 10.1002/jso.27990

**Published:** 2024-11-13

**Authors:** Nicholas J. Kelly, Neha Shafique, Gabriella N. Tortorello, Gracia Vargas, John T. Miura, Giorgos C. Karakousis

**Affiliations:** ^1^ Department of Surgery University of Pennsylvania Philadelphia Pennsylvania USA

**Keywords:** gastric cancer, morbidity, mortality, NSQIP, total gastrectomy, trends

## Abstract

**Introduction:**

We examined trends in major morbidity and mortality following total gastrectomy for malignancy in a national cohort.

**Methods:**

The National Surgical Quality Improvement Program was used to identify patients who underwent total gastrectomy for malignancy from 2007 to 2021. Joinpoint regression was used to determine annual percent changes (APCs) in thirty‐day postoperative major morbidity, mortality, and length of stay (LOS). Major morbidity included deep and organ space surgical site infection, venous thromboembolism, cardiac event, pneumonia, acute renal failure, sepsis, and respiratory failure.

**Results:**

Of 3515 patients, the median age was 65 years (IQR = 55–73), 59% were male, and 57.9% were White. Major morbidity was 23%, which did not change over time (APC = −1.4, 95% CI = −3.4 to 0.58), nor were there changes in individual morbidities with time. The most common morbidities were organ space surgical site infection (9.2%) and pneumonia (8.5%). Mortality rate in the study cohort was 2.7% and did not change (APC = −6.2, 95% CI = −13.0 to 1.1). LOS (median 9 days) also did not vary with time (APC = −2.3, 95% CI = −7.8 to 3.9). There was an increase in patients with diabetes (21.6% vs. 11.2%, *p* < 0.05), BMI ≥ 30 (31.1% vs. 18.2%, *p* < 0.05), and ASA IV–V status (11.6% vs. 3.5%, *p* < 0.05).

**Conclusion:**

Morbidity and mortality following total gastrectomy for malignancy have not significantly changed over the last fifteen years. While this may in part be explained by increased patient comorbidity, efforts should be made to improve patient selection and mitigate postoperative complications to allow for timely adjuvant therapies.

## Summary

Morbidity and mortality following total gastrectomy for malignancy have not significantly changed over the last fifteen years. This may in part be explained by a change in patient selection, with an observed increase in patients with diabetes, obesity, and poor cardiopulmonary function. These data can inform preoperative counseling for patients and identify strategies to mitigate postoperative complications to allow for timely initiation of adjuvant therapies.

## 1 Introduction

Gastric cancer is the third‐leading cause of cancer‐related death globally [1]. Total gastrectomy has historically carried substantial morbidity and mortality, driven largely by infectious and respiratory complications [2]. Prior studies have documented rates of thirty‐day major morbidity following total gastrectomy to be as high as 36% [3]. Rates of mortality, meanwhile, have been reported to be near 5%, which exceeds the mortality of major hepatectomy, pancreaticoduodenectomy, and esophagectomy [2, 3]. With an increasing focus on surgical quality and outcomes, we sought to study temporal trends in major morbidity and mortality following total gastrectomy for malignancy in a national cohort.

The National Surgical Quality Improvement Program (NSQIP) is a validated and risk‐adjusted database. It includes over 700 participating centers that prospectively collect and submit standardized thirty‐day postoperative outcome metrics [4]. Since its inception, the database has led to quality improvement initiatives across U.S. hospitals [5, 6].

Prior studies have employed the ACS‐NSQIP database to document trends in outcomes and practices for patients undergoing various types of major cancer operations, including colectomy, esophagectomy, hepatectomy, pancreatectomy, and proctectomy, but total gastrectomy for gastric malignancy remains understudied [7]. Understanding these metrics can help guide patients' subsequent treatment courses and ultimately affect their long‐term oncologic prognoses. Adjuvant chemotherapy has been shown to improve disease‐specific and overall survival for patients with gastric cancer, but the postoperative morbidity associated with total gastrectomy can delay the timely initiation of such therapies [8, 9]. Although prior studies have employed the NSQIP database to identify perioperative risk factors associated with total gastrectomy, few studies have trended outcomes, practices, and patient selection over time [3, 10].

## 2 Materials and Methods

The ACS‐NSQIP participant user file (PUF) was used to identify patients who underwent total gastrectomy for gastric malignancy from 2007 to 2021. Patients with gastric malignancy were first identified (ICD‐10 codes C16‐C16.9, C49.A2, and C7A.092; ICD‐9 codes 151‐151.9, and 171.5, 209.23). Patients who underwent total gastrectomy were then identified using Current Procedural Terminology (CPT) codes (43620 for a total gastrectomy with esophagoenterostomy, 43621 for a total gastrectomy with roux‐en‐y reconstruction, and 43622 for total gastrectomy with formation of intestinal pouch). Patients who required preoperative admission (defined as > 0 days between hospital admission and surgery), had their operations in outpatient facilities, or had disseminated cancer were excluded from the analysis. This study was exempt from approval by the University of Pennsylvania Institutional Review Board as the ACS‐NSQIP PUF includes only deidentified data.

The primary outcomes studied were thirty‐day mortality and major morbidity. Major morbidity comprised any one of the following complications: deep space surgical site infection (involving muscle/fascia), organ space surgical site infection (deep to the fascia), venous thromboembolism, cardiac event, pneumonia, acute renal failure, sepsis, and respiratory failure (intubation for ≥ 48 hours or reintubation). These major morbidities were subsequently combined to create a composite overall morbidity variable. Secondary outcomes were operative time and length of hospital stay (LOS).

Patient clinical characteristics and laboratory values, which have been demonstrated to affect morbidity and mortality following total gastrectomy, were controlled for in the analysis [11, 12]. Preoperative clinical characteristics included age (dichotomized as < 65 or ≥ 65 years of age), sex, race, ASA class (I–II, III, or IV–V), obesity (body mass index [BMI] ≥ 30 kg/m^2^), hypertension requiring at least one medication, smoking, chronic obstructive pulmonary disease, congestive heart failure, dialysis dependence, ascites, steroid use within 30 days, and weight loss (defined as loss of > 10% body weight in the 6 months before surgery). Preoperative laboratory values, which were dichotomized into categorical variables, included: creatinine (Cr < or ≥ 2 mg/dL), white blood cell count (WBC < or ≥ 12,000 cells/mcl), albumin (< or ≥ 3.5 g/dL), and total bilirubin (< or ≥ 1 mg/dL). Statistical analyses were conducted using Stata version 17 (StataCorp LLC). The National Cancer Institute's Joinpoint Regression Analysis Program was used to determine annual percent changes (APCs) in thirty‐day postoperative major morbidity, mortality, and LOS over time, and two‐sided t‐tests were used to evaluate these changes for statistical significance. The threshold for statistical significance was set at *p* < 0.05.

## 3 Results

### 3.1 Patient Demographics and Comorbidities

Of 3,515 patients who met inclusion criteria, the median age of the cohort was 65 years (interquartile range [IQR] 55–73). The majority (59%) of patients were male, and 57.9% were White. The majority of patients were ASA class III (67.5%). 16.8% of patients had diabetes, 25.8% of patients had a BMI ≥ 30, and 48.8% of patients had hypertension requiring at least one medication. 16.2% of patients were current smokers or had smoked within 1 year before their procedure. Descriptive characteristics of the patient cohort are presented in Table 1.

**Table 1:** Patient demographics and clinical characteristics.

Factor	No. (%)
**Median Age (years, median, IQR)**	65 (55–73)
< 65	1657 (47.1%)
≥ 65	1858 (52.9%)
**Male**	2094 (59.6%)
**Race**	
White	2134 (60.8%)
Black	418 (11.9%)
Asian American Pacific Islander	363 (10.3%)
Other/Unknown	596 (17.0%)
**ASA Class**	
I–II	956 (27.3%)
III	2365 (67.5%)
IV–V	185 (5.3%)
**BMI**	
< 30	2609 (74.2%)
≥ 30	906 (25.8%)
**Hypertension**	1716 (48.8%)
**Current smoker**	569 (16.2%)
**COPD**	123 (3.5%)
**CHF**	18 (0.5%)
**Diabetes**	591 (16.8%)
**Dialysis**	9 (0.3%)
**Ascites**	8 (0.2%)
**Steroid use within 30 Days**	112 (3.2%)
**Weight loss**	441 (13.1%)
**Creatinine** > **2**	171 (4.9%)
**WBC** ≥ **12**	204 (5.8%)
**Albumin** < **3.5**	498 (14.2%)
**Bilirubin** ≥ **1**	827 (23.5%)

Abbreviations: ASA, American Society of Anesthesiology; BMI, body mass index; CHF, congestive heart failure; COPD, chronic obstructive pulmonary disease; IQR, interquartile range; WBC, white blood cell.

Over the study period, there was an increase in the percentage of patients with diabetes from 2007 to 2021 (21.6% vs. 11.2%, *p* < 0.05), BMI ≥ 30 (31.1% vs. 18.2%, *p* < 0.05), and ASA IV–V status (11.6% vs. 3.5%, *p* < 0.05). There was also a decrease in percentage of patients > 65 years of age, although this was not statistically significant (47.3% vs 58.0%, *p* = 0.06). There were otherwise no significant changes in patient clinical or laboratory characteristics over the study period.

### 3.2 Outcomes Among Patients Undergoing Total Gastrectomy Over Time

**Figure 1:**
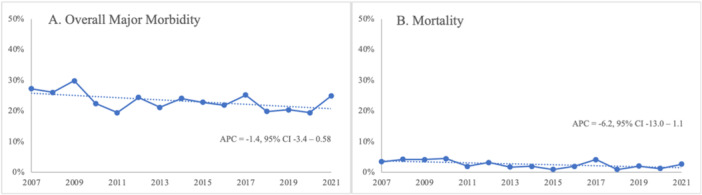
(A) 30‐day overall major morbidity and (B) Mortality after total gastrectomy between 2007 and 2021.

**Figure 2:**
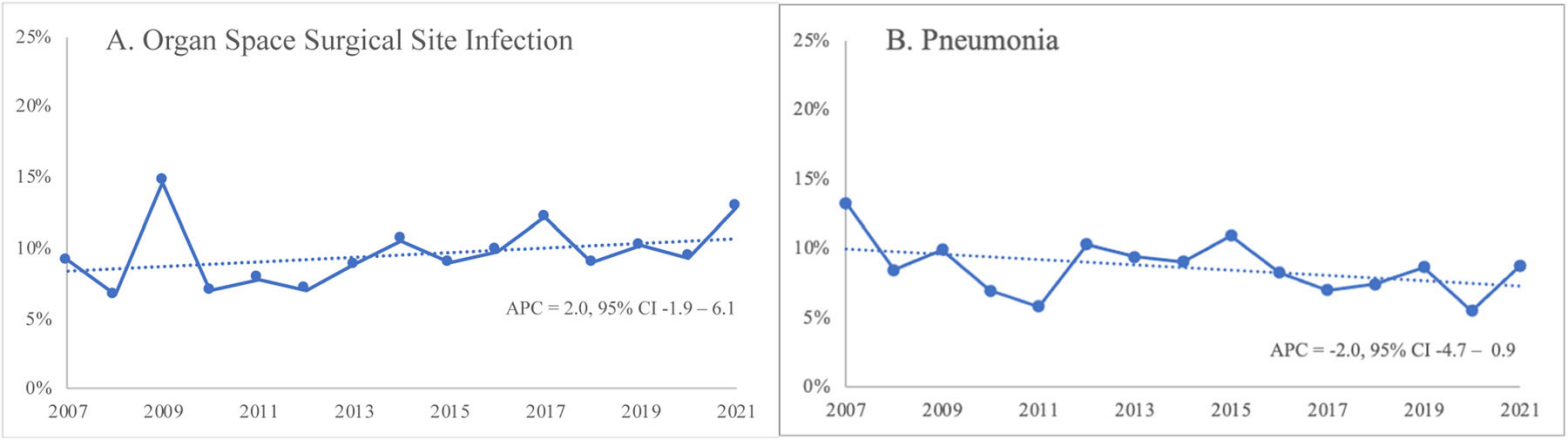
Most common morbidities between 2007 and 2021. (A) Organ space surgical site infection. (B) Pneumonia.

Overall 30‐day major morbidity, our composite measure of morbidity, was 23%, and this did not change significantly over time (APC = −1.4, 95% CI = −3.4 to 0.58; 27.3% in 2007 to 25.0% in 2021) (Figure 1A). Individual postoperative morbidities did not change significantly over the study period. This included organ space surgical site infection (APC = 2.0, 95% CI = −1.9 to 6.1; 9.1% in 2007 to 12.8% in 2021). The distribution of postoperative complications is displayed in Table 2. The most common morbidities were organ space surgical site infection (9.2%) and pneumonia (8.5%), and these are displayed over time in Figure 2. Among patients who developed a complication, nearly half (47.3%) developed two or more major complications.

**Table 2:** Distribution of morbidities.

Morbidity	No. (%)
**Overall morbidity**	807 (23.0%)
**Organ space surgical site infection**	324 (9.2%)
**Pneumonia**	298 (8.5%)
**Sepsis**	267 (7.6%)
**Respiratory failure**	240 (6.8%)
**Venous thromboembolism**	98 (2.8%)
**Myocardial infarction/cardiac arrest**	67 (1.9%)
**Deep space surgical site infection**	48 (1.4%)
**Acute renal failure**	30 (0.9%)

**Figure 3:**
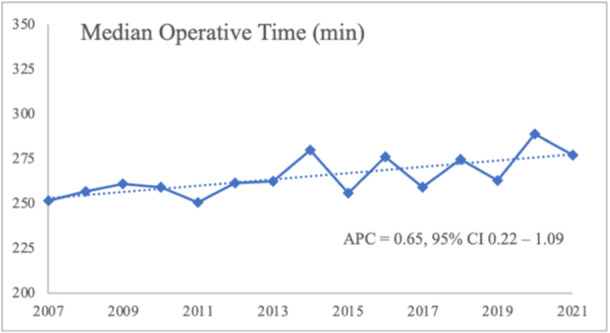
Median operative time.

The mortality rate in the study cohort was 2.7% and did not significantly change between 2007 and 2021 (APC = −6.2, 95% CI = −13.0 to 1.1) (3.5% in 2007 to 2.7% in 2021) (Figure 1B). Median LOS was 9 days and also did not vary significantly with time (APC = −2.3, 95% CI = −7.8 to 3.9). The median operative time showed a small but significant increase over time from 252 to 277 min (APC = 0.65, 95% CI = 0.22–1.09) (Figure 3).

## 4 Discussion

Major morbidity and mortality following total gastrectomy for malignancy did not change significantly over the 15 years examined in the current study. There was, however, a change in patient comorbidities over time. Specifically, there was an upward trend in patients with diabetes, BMI ≥ 30, and elevated ASA class undergoing total gastrectomy over time, which may reflect expanding patient eligibility [13]. The patient characteristics that changed significantly in the current study are well documented risk factors in the literature. A recent systematic review of 16 studies involving 42,489 patients who underwent total gastrectomy for gastric cancer identified significant associations between diabetes mellitus, obesity (BMI and visceral fat area), and respiratory function (ASA class) with anastomotic leakage after esophago‐jejunostomy [13]. Anastomotic leakage, which has been reported to occur at rates of 2–15% for total gastrectomy, may drive many of the other complications (e.g., mortality, length of stay, sepsis). While we are unable to assess anastomotic leak directly as this is not specifically collected in the NSQIP PUF, organ space infection may be used as a proxy. The rates of organ space infection along with any resultant comorbidities did not show any change over time during our study period. Complication rates remaining steady despite increasing medical comorbidity of patients may in fact indirectly reflect progress in surgical quality over time.

Infectious and respiratory complications were the most common postoperative complications seen in our study. This could help identify areas of improvement with consistent adherence to routine preventive protocols, such as pulmonary toilet, suction tube placement to prevent aspiration, and/or effective pain management and use of incentive spirometry to help prevent postoperative pneumonia secondary to atelectasis. Recognizing these complications are the most common can also inform preoperative counseling for the highest‐risk patient subgroups, such as those with a significant smoking history or those who are immunocompromised.

There are several notable limitations to the current study results. Firstly, the chemotherapy and radiation fields were not recorded in the ACS‐NSQIP database after 2012. Perioperative chemotherapy and adjuvant chemoradiation have been associated with increased disease‐specific and overall survival [3]. The MAGIC trial found that, compared to patients who received surgery alone, patients who received both surgery and chemotherapy had smaller and less advanced tumors as well as improved progression‐free and overall survival [14]. The French‐based FFCD/FNCLCC trial, whose study population included a larger proportion of patients with tumors in the lower esophagus and gastroesophageal junction, produced similar results: patients who received a perioperative chemotherapy regimen of cisplatin and fluorouracil had significantly improved overall and disease‐specific survival than patients who received surgery alone [15]. The German‐based FLOT4 trial demonstrated that patients who received a regimen of FLOT (fluorouracil plus leucovorin, oxaliplatin, and docetaxel) had lower postoperative T‐stage and N‐stage as well as higher rates of margin‐free resection than patients receiving therapy with ECF/ECX (epirubicin and cisplatin plus either fluorouracil or capecitabine), which translated to improved overall survival for the FLOT group [16]. These studies suggest that smaller, less advanced tumors could theoretically simplify the operation, leading to improved morbidity and mortality. Conversely, preoperative treatment with chemotherapy or radiation could be associated with higher risk of surgical complications by causing inflammation and fibrosis, leading to issues with anastomotic or wound healing [17, 18]. As our study is unable to stratify patients based on their receipt of neoadjuvant therapy or disease stage, we are unable to control for these possible competing effects.

Another limitation is our inability to discern surgical technique. Specifically, the ACS‐NSQIP database does not include information on surgical approach for these years, and there is no CPT code that indicates laparoscopic total gastrectomy, precluding our ability to compare minimally invasive versus open approaches. We did observe a small but significant increase in median operative time over the study period, which may reflect increasing adoption of minimally invasive approaches that have been previously shown to have longer operating times [19]. Although the literature on morbidity and mortality following laparoscopic versus open total gastrectomy remains inconclusive, some studies have suggested that laparoscopic total gastrectomy may confer advantages over the open approach, including a lower complication rate, fewer wound infections, and shorter hospital stays [20, 21]. An increasing proportion of cases performed via a minimally invasive approach may have influenced the study results though morbidities are likely driven from the physiologic demands of a total gastrectomy regardless of surgical approach. Further research is needed to trend outcomes of laparoscopic versus open total gastrectomy over time. A final limitation of the current analysis pertains to the relatively short‐term nature of the NSQIP outcome measures themselves, reported only until postoperative day 30. One study that examined 90‐day mortality after total gastrectomy for gastric cancer noted that more than half of the study's deaths occurred beyond postoperative day 30 [22].

## 5 Conclusion

In conclusion, 30‐day major morbidity and mortality, as well as length of hospital stay, have remained relatively unchanged in patients who received total gastrectomy for gastric malignancy over the last 15 years. This lack of demonstrable improvement over time may be explained in part to changes in patient selection. Indeed, advances in pre and postoperative management along with surgical technique may be offset by an increasing proportion of patients with diabetes, obesity, and poor respiratory function as well as patients with more advanced disease. Further studies could: (1) continue to investigate these trends as data on laparoscopic versus open repair become more readily available; and (2) evaluate longer‐term outcomes. Meanwhile, continued efforts should be made to improve patient selection and mitigate postoperative complications to allow for timely initiation of adjuvant therapies.

## Conflicts of Interest

No authors declare conflicts of interest.

## 6 Synopsis

In this national cohort study, major morbidity and mortality following total gastrectomy for malignancy were found to have remained relatively stable over the last 15 years despite an increasingly comorbid patient population.

## Data Availability

Data subject to third party restrictions. The data that support the findings of this study are available from the ACS NSQIP Participant User File. Restrictions apply to the availability of these data, which were used under license for this study. Data are available at https://www.facs.org/quality-programs/data-and-registries/acs-nsqip/participant-use-data-file/ with the permission of ACS NSQIP.
